# How often do oncologists receive industry payments from competing companies?

**DOI:** 10.1093/oncolo/oyag002

**Published:** 2026-01-09

**Authors:** Helen M Keetley, Ziad Zakaria, Grace Gallagher, Genevieve P Kanter, Aaron P Mitchell

**Affiliations:** Department of Epidemiology and Biostatistics, Memorial Sloan Kettering Cancer Center, New York, NY 10017, United States; Department of Epidemiology and Biostatistics, Memorial Sloan Kettering Cancer Center, New York, NY 10017, United States; Department of Epidemiology and Biostatistics, Memorial Sloan Kettering Cancer Center, New York, NY 10017, United States; Department of Health Policy and Management, Sol Price School of Public Policy, University of Southern California, Los Angeles, CA 90089, United States; Schaeffer Center for Health Policy and Economics, University of Southern California, Los Angeles, CA 90089, United States; Department of Epidemiology and Biostatistics, Memorial Sloan Kettering Cancer Center, New York, NY 10017, United States; Department of Medicine, Division of Solid Tumor Oncology, Memorial Sloan Kettering Cancer Center, New York, NY 10065, United States

**Keywords:** industry payments, pharmaceutical industry, prescribing practice, practice patterns, antineoplastic agents, health policy, drug promotion

## Abstract

**Background:**

Payments from pharmaceutical companies to oncologists can influence prescribing practices. However, some physicians believe that receiving payments from multiple competing manufacturers might balance these biasing effects, effectively “canceling out” any conflict of interest. This study examines how often physicians receive industry payments for multiple, competing drugs.

**Methods:**

Using the CMS Part D Prescribers file 2017-2019, we included medical oncologists who prescribed a class of cancer drugs wherein there are multiple, competing drugs. We then matched these oncologists to their industry payments records (Open Payments). We assessed the proportion of oncologists who received any industry payments related to 0, 1, 2, or 3 of the competing drugs, and whether oncologists prescribed differently with respect to the number of drugs for which they received payment.

**Results:**

Among 2460 eligible oncologists, a minority received payments related to all 3 competing drugs within the drug class they prescribed: 1.6% of oncologists prescribing epidermal growth factor receptor inhibitors, 25.3% for BCR-ABL tyrosine kinase inhibitors, and 34.3% for CDK4/6 inhibitors. Oncologists who received payments for all 3 drugs were more likely to prescribe ribociclib versus palbociclib, and less likely to prescribe dasatinib, compared to unpaid oncologists. Those who received payments for 2 drugs were more likely to prescribe afatinib versus osimertinib.

**Conclusions:**

Receipt of payments from all competing companies occurs among a minority of oncologists, but a substantial minority for some drug classes. Oncologists who receive payments from multiple companies have different prescribing patterns than unpaid oncologists, suggesting that competing payments may not result in “balanced” influence.

Implications for Practice We aimed to evaluate whether oncologists’ receipt of promotional payments from multiple competing drug companies may “balance” bias on prescribing. Within a 3-year period, the proportion of oncologists who received any payments from all competing manufacturers ranged from 1.6% for epidermal growth factor receptor inhibitors to 34.3% for CDK4/6 inhibitors. However, oncologists who received payments from multiple companies had different prescribing patterns from those who remained unpaid. These findings suggest that payments from competing drug manufacturers may not result in fully “balanced bias” among the substantial minority of oncologists who receive such payments.

## Introduction

Financial payments from the pharmaceutical industry affect physicians’ prescribing practices. Receipt of pharmaceutical company payments for a promoted drug is associated with increased prescribing of that drug over competitors.^1^ This association has been observed for several classes of cancer drugs, including epidermal growth factor receptor inhibitors (EGFRs), Vascular Endothelial Growth Factor Tyrosine Kinase Inhibitors, Breakpoint Cluster Region-Abelson murine Leukemia (BCR-ABL) TKIs, anti–androgens, and PARP inhibitors.^2–5^ There is strong evidence that the relationship between payments and prescribing is causal, not merely correlational.^6^^,^^7^ Interactions with pharmaceutical companies have also been associated with low-quality prescribing, such as an increase in prescriptions of non–recommended or low-value treatments.^8–10^

A rationale presented by some physicians in defense of receiving industry money and gifts is that payments from competing companies “cancel each other out” in terms of any potential biasing effect on prescribing. In other words, receiving payments from multiple competing companies is balancing and negates any biasing effects these payments may have had.^11^ There is some empirical support for this claim. In the context of FDA advisory committees, members who had relationships with only 1 company appear to be biased; however, this bias appeared to be mitigated by having relationships with multiple, competing companies.^12^

The potential for biased prescribing is especially relevant to oncology. Not only is it among the specialties that receive the highest dollar value of industry payments,^13^ but there are large differences in the degree of clinical benefit among treatment options, even among guideline-recommended treatments.^14^^,^^15^ Biased prescribing is most concerning when it has the potential to increase the use of less-optimal agents. For example, among the tyrosine kinase inhibitors used for chronic myeloid leukemia (CML), industry payments have shifted physicians toward dasatinib and nilotinib and away from imatinib despite their greater toxicity.^2^^,^^16^

While this argument for the balancing effects of competing payments appears to be plausible, it is unknown how often this actually occurs in real-world clinical practice. For this potential “balancing” effect to be relevant to the overall landscape of physician-industry COI, it would be necessary for it to occur among a substantial portion of physicians. Prior work has found that oncologists often receive money from manufacturers of multiple immunotherapy drugs, which have broad applications across multiple cancer types.^17^ However, whether this also the case for oral targeted therapies used for a narrower set of cancer types is unknown.

## Methods

### Study design

We studied receipt of industry payments using Open Payments, a public database of transfers of financial value (“payments”) from drug and device manufacturers to US health care providers, during 2017-2019. We chose a study period of 3 years’ duration to reflect the amount of time in which, if a physician received payments from multiple competing companies, the payments might conceivably “balance each other” in terms of influence on physician prescribing. Prior work has suggested that the biasing effect of an industry payment may last slightly longer than 1 year;^6^ we therefore chose a 3-year study period to allow for the possibility that a payment received at the end of calendar year (1) might still be exerting some influence during subsequent calendar year (3). We selected the specific 3-year period of 2017-2019 because it was the most recent 3-year period available in Open Payments data that did not overlap with the COVID-19 pandemic, to avoid our data being influenced by pandemic-related disruptions in industry payment patterns.^18^

We identified cases during our 2017-2019 study period where there were multiple, substitutable drugs within the same class that are all treatment options for the same cancer type. For such a “choice set” of drugs to be included, we required each drug to be FDA-approved and NCCN-recommended for the same cancer type across the study period. Of note, the requirement for NCCN recommendation does not imply that all drugs within a choice set are clinically equivalent, because the NCCN commonly recommends multiple drugs with a range of efficacy and toxicity profiles. In order to ensure that physicians had equal opportunity to receive payments for all drugs in a choice set, we required that drugs were FDA-approved during each year of the study period (avoiding the possibility of physicians having 3 years to accrue payments for some drugs and less time for others). For similar reasons, we excluded drugs that became available as generics during the study period, because pharmaceutical companies stop promotional payments for drugs with generic competitors.^6^ Coincidentally, all 6 choice sets meeting these requirements had exactly 3 drugs: renal cell carcinoma (RCC, consisting of sunitinib, sorafenib, and pazopanib); CML: dasatinib, nilotinib, bosutinib; epidermal growth factor receptor inhibitors (EGFRs: afatinib, gefitinib, osimertinib); anaplastic lymphoma kinase inhibitors (ALK: alectinib, ceritinib, crizotinib); poly ADP ribose polymerase inhibitors (PARP: Olaparib, rucaparib, niraparib); and cyclin dependent kinase 4/6 inhibitors (CDK: Palbociclib, ribociclib, abemaciclib).

### Physician prescriber cohort

We used the CMS Part D: Prescribers file to identify our physician sample. We included physicians with a specialty of Hematology/Oncology or Medical Oncology who also had records in the Doctors and Clinicians National Downloadable File (linked by NPI), which reports physician characteristics. We then defined a physician sub–cohort corresponding to each choice set of drugs, consisting of physicians who consistently prescribed drugs in that class. Physicians were included in a sub–cohort if they prescribed 1 or more of the drugs in that choice set during each year from 2017 to 2019 (eg, in 3 consecutive years). Physicians could be present in more than 1 sub–cohort if they met the prescribing requirements of each. In order to ensure a sufficient number of physicians for the analysis to be informative, we retained sub–cohorts with at least 100 physicians and excluded those with fewer.

To measure industry payments, we matched physician data to Open Payments data by NPI. Physicians without Open Payments records were assumed to have received no industry payments. General payments were attributed to the physician listed as recipient, and research payments were attributed to the physician[s] listed as principal investigator. Because of the low prevalence of ownership interests, we excluded this payment type from analysis.

We calculated physician prescribing volume in terms of 30-day fills. We used this volume measure to characterize physician prescribing and to identify high-volume prescribers within each sub–cohort. We define high-volume prescribers as physicians whose prescribing volume was in the top quartile of 30-day fills across all 3 drugs in each choice set over the full 2017-2019 study period. Top prescribers are specific to each choice set (sub–cohort) so that, for example, a physician who was in both the CML and EGFR sub–cohorts might be a top-quartile prescriber of CML drugs but not EGFR drugs.

### Analysis

The primary analysis was the proportion of physicians who received any industry payments related to 0, 1, 2, or all 3 of the competing drugs during the study period. In addition to analyzing receipt of *any* payment, we also explored receipt of payments that may indicate more substantial relationships with industry. Alternative types of payments we analyzed included: (1) payments totaling $100 or more, (2) more than 1 payment, and (3) any non–food and beverage payment. Food and beverage payments are the most common kind of payment and some do not consider these to represent “true” conflicts of interest; for example, the NCCN excludes food and beverage payments from their conflict-of-interest policy.^19^^,^^20^ We therefore chose the >$100 cutoff and the non–food and beverage requirement as thresholds that would exclude the majority of physicians who received only low-value food and beverage payments.

We also compared physicians who had received payments for 0, 1, 2, or all 3 drugs, by physician characteristics: gender, years since medical school graduation (proxy for career stage), and high-volume prescribing. We assessed whether differences in the distribution of these characteristics were statistically significantly different using Chi-squared tests. To assess whether physicians who received payments for more drugs prescribed differently than those who received payments for fewer drugs, we compared prescription count and the proportion of prescriptions for each drug within a choice set using ANOVA.

## Results

We identified 13 827 physicians with a specialty of Hematology Oncology or Medical Oncology in the Part D prescriber file (Figure S1). After applying the additional eligibility requirements, we found that fewer than 100 prescribers remained within the RCC, ALK, and PARP choice sets, which were therefore dropped from analysis. For the remaining CML, EGFR, and CDK choice sets, we identified 2460 unique physicians who were consistent prescribers of one or more of these choice sets: CML, *n* = 810; EGFR, *n* = 243; CDK, *n* = 1756 (Table 1). Of these physicians, 1653 (67.2%) were men and 807 (32.8%) were women. Only 24 (1.0%) had fewer than 10 years since medical school graduation, and 850 (34.6%) had more than 30 years since graduation (Table 1). Within each choice set, there was substantial variation in prescribing volume among the 3 drugs; some drugs were prescribed substantially more frequently than others (Table S1). The drug class with the greatest relative difference between the most-prescribed and least-prescribed drug was CDK4/6 inhibitors, with a per-physician mean of 32.4 30-day fills for palbociclib versus 0.7 for ribociclib.

**Table 1.** Physician characteristics.

Characteristic	Overall *N* = 2460 (100%)
**Gender**	
** Woman**	807 (32.8)
** Man**	1653 (67.2)
**Years since graduation**	
** <10 years**	24 (1.0)
** 10-20 years**	790 (32.2)
** 21-30 years**	792 (32.3)
** 30+ years**	850 (34.6)
** Unknown**	4
**Sub–cohort**	
** CML**	810 (32.9)
** EGFR**	243 (9.9)
** CDK**	1756 (71.4)

Sub–cohort totals sum to greater than the overall cohort size (*N* = 2460) because physicians could be included in more than 1 sub–cohort. Abbreviations: CDK, cyclin dependent kinase 4/6 inhibitors; CML, chronic myeloid leukemia; EGFR, epidermal growth factor receptor inhibitors.

There was wide variation among physicians regarding industry payments received. The mean dollar value of general payments received related to the drugs of interest during 2017-2019 ranged from $800 for CML to $6923 for EGFR (Table 2). The mean dollar value of research payments ranged from $52 460 for CDK4/6 inhibitors to $104 453 for EGFR drugs. Payments also varied substantially among the drugs in each choice set. The mean dollar value of general payments for each drug ranged from $1 for gefitinib to $5398 for osimertinib. The mean dollar value of research payments for each drug ranged from $0 for abemaciclib in the CDK4/6 cohort to $100 350 for osimertinib in the EGFR cohort.

**Table 2.** Industry payments received by physicians.

	CML			EGFR			CDK		
**Physician *N***	810			243			1756		
**Mean value, GP (SD) **	800 (5942)			6923 (19 702)			2653 (18 380)		
**Median value, GP (IQR)**	47 (214)			72 (3029)			78 (355)		
**Mean value, RP (SD) **	86 423 (1 658 335)			104 453 (1 174 670)			52 460 (565 420)		
**Median value, RP (IQR)**	0 (0)			0 (0)			0 (0)		
**Drug **	**Dasatinib**	**Nilotinib**	**Bosutinib**	**Afatinib**	**Gefitinib**	**Osimertinib**	**Palbociclib**	**Ribociclib**	**Abemaciclib**
**Mean value of GP (SD) **	223 (3469)	438 (3653)	140 (2274)	1524 (9191)	1 (6)	5398 (16 940)	582 (3461)	1278 (11 561)	794 (8349)
**Median value of GP (IQR) **	0 (15)	18 (131)	0 (53)	0 (154)	0 (0)	14 (521)	15 (124)	16 (106)	0 (59)
**Mean value of RP (SD) **	75 441 (1 517 906)	2965 (53 375)	8017 (176 495)	3079 (23 384)	1024 (14 500)	100 350 (1 174 702)	29 698 (521 222)	22 763 (191 081)	0 (0)
**Median value of RP (IQR) **	0 (0)	0 (0)	0 (0)	0 (0)	0 (0)	0 (0)	0 (0)	0 (0)	0 (0)
**Mean total dollar value, GP** **+** **RP (SD)**	**Dasatinib**	**Nilotinib**	**Bosutinib**	**Afatinib**	**Gefitinib**	**Osimertinib**	**Palbociclib**	**Ribociclib**	**Abemaciclib**
** 2017 **	20 019 (398 457)	2637 (48 045)	3699 (80 335)	1619 (15 499)	579 (8383)	30 952 (411 197)	10 314 (201 749)	8727 (71 210)	5 (13)
** 2018 **	22 334 (463 691)	473 (5923)	2635 (59 884)	2362 (15 657)	97 (1206)	46 950 (538 660)	9389 (143 114)	8650 (77 360)	310 (3510)
** 2019 **	33 310 (667 118)	293 (2248)	1822 (38 826)	622 (3512)	350 (4947)	27 846 (248 945)	10 575 (197 158)	6664 (54 101)	479 (5132)

The per-physician dollar value of payments received, during the full 2017-2019 study period, is reported for each sub–cohort and payment type. Mean and median values are reported in nominal USD. Values reflect only payments related to 1 of the 3 drugs in the relevant choice set, and do not include industry payments related to any other drugs. Mean and median payment values are also shown for each of the 3 drugs individually. Abbreviations: CDK, cyclin dependent kinase 4/6 inhibitors; CML, chronic myeloid leukemia; EGFR, epidermal growth factor receptor inhibitors; GP, general payment; IQR, interquartile range; RP, research payment; SD, standard deviation.

Assessing total payment amounts (general and research) received related to the drugs in the corresponding choice set, we found that payments followed a bimodal distribution across physicians (Figure S2). Most physicians received less than $1000 total related to the drugs of interest, but a smaller proportion received substantially larger amounts typically ranging from $10 000-$100 000; this bimodal distribution was most apparent within the EGFR sub–cohort. This bimodal distribution remained apparent when aggregating physicians across all 3 sub–cohorts (Figure S3).

**Figure 1.**
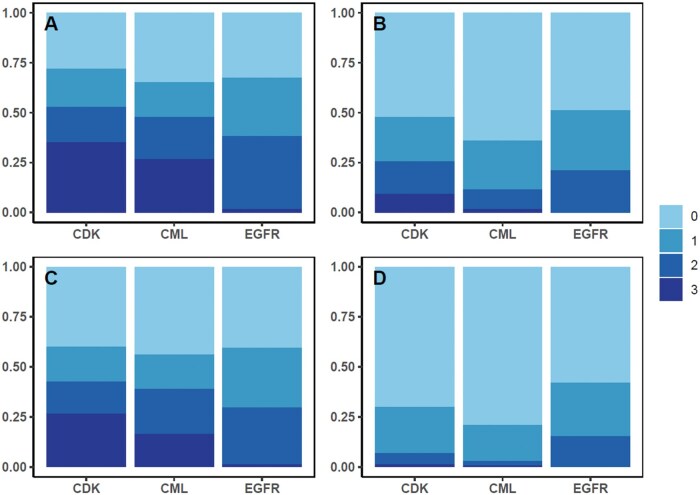
Proportion of physicians within each drug sub–cohort who received payments for 0, 1, 2, or 3 of the competing drugs in that class. (A) Physicians who received any general or research payment were counted as having received payment. (B) Physicians who received payments totaling $100 or greater were counted as having received payments. (C) Physicians who received 2 or more payments were counted as having received payments. (D) Physicians who received any payments other than food and beverage payments were counted as having received payments. CDK, cyclin dependent kinase 4/6 inhibitors; CML, chronic myeloid leukemia; EGFR, epidermal growth factor receptor inhibitors.

Within all sub–cohorts, only a minority of physicians had received any payments related to all 3 competing drugs (Figure 1, Table S2). When including both general and research payments, this proportion was highest among the CDK4/6 inhibitor choice set at 34.3% of physicians and lowest within EGFR (1.6%). The proportion of physicians who received payments for *1 or 2* competing drugs (eg, the physicians who received payments that were “unbalanced” across all competing drugs) was 37.0% (649 of 1756) for CDK4/6 inhibitors, 38.5% (312 of 810) for CML, and 65.8% (160 of 243) for EGFR. Finally, the proportion of physicians who did not receive any payments related to the drugs of interest was 28.0% (491 of 1756) for CDK4/6 inhibitors, 32.5% (79 of 243) for EGFR, and 34.9% (283 of 810) for CML.

When assessing whether physicians had received payments totaling $100 or more, more than 1 payment, or non–food and beverage payments, for a given drug, the proportion of physicians who received such payments for all 3 drugs within a choice set was substantially lower. Using CDK4/6 inhibitors (the choice set with the highest proportion receiving payments for all 3 drugs) as an example case, the proportion receiving payments for all 3 drugs was reduced from 35.1% to 9.2% when assessing payments totally $100 or more (Table S3), to 26.5% when assessing receipt of more than 1 payment (Table S4), and to 1.3% when assessing receipt of non–food and beverage payments (Table S5).

Physicians who received payments related to a greater number of drugs had different characteristics from those who received payments related to fewer drugs (Table 3). Men made up 66.4% of physicians who received payments for zero drugs and 74.9% of physicians who received payments for all 3 drugs (*P* < 0.001). Top-quartile prescribers were 17.4% of those who received payments for 0 drugs and 27.2% of those who received payments for all 3 drugs (*P* < 0.001). Time since medical school graduation was not statistically significantly different with respect to number of paid drugs. These trends were similar within each sub–cohort individually (Tables S6-S8).

**Table 3.** Distribution of physician characteristics with respect to number of drugs for which the physician received payment.

	Number of drugs for which physician received any industry payments, *n* (%)				*P*-value
	0	1	2	3	
**Physician total**	853	548	573	835	
**Gender **					<.001
** Woman **	287 (33.6)	212 (38.7)	172 (30.0)	210 (25.1)	
** Man **	566 (66.4)	336 (61.3)	401 (70.0)	625 (74.9)	
**Years since graduation **					.19
** <10 years **	7 (0.8)	5 (0.9)	3 (0.5)	9 (1.1)	
** 10-20 years **	299 (35.1)	177 (32.3)	169 (29.6)	244 (29.2)	
** 21-30 years **	267 (31.4)	183 (33.4)	197 (34.6)	263 (31.5)	
** 30+ years**	278 (32.7)	183 (33.4)	201 (35.3)	319 (38.2)	
** Unknown**	2	0	3	0	
**Prescribing volume**					<.001
** Top quartile prescribers**	148 (17.4)	155 (28.3)	177 (30.9)	227 (27.2)	
** All other prescribers **	705 (82.6)	393 (71.7)	396 (69.1)	608 (72.8)	

Results are shown for all 3 sub–cohorts in aggregate, with the unit of analysis being the physician-sub–cohort pair; physicians could be present in more than 1 sub–cohort. Chi-squared *P*-values were assessed for each characteristic in aggregate.

Although results varied by sub–cohort, in many cases physicians who received payments for multiple drugs prescribed differently than those who did not receive payments (Table 4). Physicians who received payments for all 3 drugs in the CML choice set prescribed relatively less dasatinib than unpaid physicians (43.0% vs 52.9%). Within the EGFR choice set, very few physicians (*n* = 4) received payments for all 3 drugs (limiting statistical comparison), but those who received payment for 2 drugs (primarily osimertinib and afatinib, see Table 2) prescribed relatively more afatinib (33.1% vs 16.1%) and relatively less osimertinib (61.0% vs 75.9%) than unpaid physicians. Within the CDK choice set, physicians who received payments for all 3 drugs prescribed relatively less palbociclib (93.5% vs 97.3%) and relatively more ribociclib (3.3% vs 0.1%) than unpaid physicians.

**Table 4.** Prescribing volume and distribution with respect to number of drugs for which the physician received payment.

	Number of drugs for which physician received any industry payments, *n* (%)			
	0	1	2	3
**CML**				
**Physicians**	283	141	171	215
**Mean prescriptions, total**	64.1	71.6	74.9	75.1
** Dasatinib, *N* (%)**	34.3 (52.9)	31.4 (39.7)*	32.3 (43.9)	31.7 (43.0)*
** Nilotinib, *N* (%)**	25.8 (41.9)	35.1 (54.0)*	35.6 (49.4)	36.7 (49.8)
** Bosutinib, *N* (%)**	4.0 (5.2)	5.2 (6.3)	7.0 (6.8)	6.7 (7.3)
**EGFR**				
**Physicians**	79	71	89	4
**Mean prescriptions, total**	81.7	100.0	107.7	71.3
** Afatinib, *N* (%)**	12.6 (16.1)	19.0 (25.9)	29.4 (33.1)**	19.5 (20.3)
** Gefitinib, *N* (%)**	5.6 (8.0)	3.0 (3.2)	5.7 (5.9)	3.0 (5.3)
** Osimertinib, *N* (%)**	63.5 (75.9)	78.0 (70.9)	72.6 (61.0)*	48.8 (74.5)
**CDK**				
**Physicians**	491	336	313	616
**Mean prescriptions, total**	91.5	105.8	108.9	106.2
** Palbociclib, *N* (%)**	88.7 (97.3)	101.1 (96.0)	103.0 (95.4)	98.7 (93.5)***
** Ribociclib, *N* (%)**	0.6 (0.1)	1.4 (1.3)	2.6 (1.9)	3.5 (3.3)***
** Abemaciclib, *N* (%)**	2.1 (2.0)	3.3 (2.8)	3.4 (2.7)	4.0 (3.2)

We used ANOVA to test for differences in total prescribing volume and the proportion of prescribing volume constituted by each drug, with respect to the number of drugs the physician received payment. For tests in which the null hypothesis of no difference was rejected (*F*-statistic *P*-value <0.05), we conducted Bonferroni post-hoc comparisons. *P*-values for comparisons vs. the 0 payments category are shown with asterisks:

* *P* < .05;

** *P* < .01;

*** *P* < .001.

## Discussion

Within each choice set of cancer drugs, we found that only a minority of physicians received industry payments for all competing drugs. This result suggests that the proposed possibility of payments for competing drugs “balancing out” bias would apply to a relatively small proportion of active prescribers. However, this proportion of physicians varied by drug class; more than 1/3 of prescribers of CDK4/6 inhibitors received payments for all 3 drugs. This is a sizeable enough proportion that a “balancing” effect of competing industry payments might have a substantial impact on overall treatment patterns.

If competing payments had a “balancing” effect on prescribing behavior, then the physicians with the greatest potential for bias would be those who received payment from *some but not all companies*; in the context of this study, those who received payments related to 1 or 2 of the competing drugs. Biased prescribing is known to occur even with small gifts or individual meals.^21–23^ It is therefore quite possible that even low dollar-value, food and beverage payments—which were the large majority of payments observed in this study—can lead to bias and, implicitly, lead to “balanced” bias when received for competing drugs. This group (physicians who received payments for 1 or 2 of the competing drugs, but not all) was about one-third of prescribers of CDK4/6 inhibitors and CML drugs, though about two-thirds of EGFR drugs (Table S2). This potential for biased prescribing is a concern in the context of treatment choices with unequal clinical benefit. For example, some EGFR inhibitor prescribers in our study received payments for afatinib only, despite the emergence of osimertinib as the superior drug for this indication.

However, we also found evidence that payments from competing companies may not fully balance out prescribing practices. Physicians who received payments related to all drugs in a choice set often prescribed differently than those who did not receive payments (Table 4). This is another potential concern in the context of treatment choices with unequal clinical benefit. For example, in our cohort of EGFR inhibitor-prescribing physicians, (because industry payments for gefitinib were rare, physicians who receive payments for 2 of the 3 drugs largely reflect those who received payments for afatinib and osimertinib) physicians who received payments for both afatinib and osimertinib prescribed relatively *more* afatinib and *less* osimertinib than physicians who received payments for neither drug. This observation suggests that afatinib payments are successful in increasing prescribing of this drug, even when counterbalanced by osimertinib payments. While more study is needed, these trends would call into question the premise of “balanced bias.”

We included research payments in addition to general payments, which have been more extensively studied. Many past studies have firmly established general payments to be biasing, including on the prescribing cancer drugs. There has been relatively little research on the impact of research payments, but that which is available suggests that research payments may also be associated with physician prescribing.^2^ We therefore included these payments as well.

As documented in multiple prior studies, we observed that the amount of industry payment received by individual oncologists was highly skewed, with most physicians receiving low-dollar payment amounts and small share of physicians receiving very large payments.^3^^,^^24^^,^^25^ In addition to the skewed distribution, we also observed bimodality. This bimodal distribution results from our inclusion of both general and research payments. Most oncologists in the sample received only general payments in the food and beverage category, which are typically of relatively low value (<$100), while a smaller portion of oncologists received any research payments, which can range as high as $100 000 s per payment.

This study is limited by a focus on oral cancer drugs and not provider-administered drugs such as chemotherapy or immunotherapy. Receiving industry payments is only 1 factor of many that influence prescribing, including clinical benefit,^26^ practice setting,^27^^,^^28^ and peer networks.^29^ Our physician selection method to identify physicians who consistently prescribed within each choice set was intended to be specific rather than sensitive; a result of this is that each sub–cohort likely disproportionately reflects disease group sub–specialists rather than generalist oncologists, who may have different patterns of industry relationships. Additionally, because the CMS prescriber file includes prescription counts only when a physician prescribes a drug 10 or more times in a given year, our cohort identification process likely excludes physicians who were consistent but infrequent prescribers of the drugs of interest. Our study period was 2017-2019, and our conclusions may not apply to other time periods.

## Conclusions

Among oncologists, a substantial minority receive financial payments from all competing manufacturers within drug classes they commonly prescribe. Payments from competing manufacturers have the potential to balance out any directional bias or influence on physician prescribing. If this occurred, then the influence of industry payments on physician prescribing may be concentrated among physicians who have received “unbalanced” payments from some but not all competing companies. However, while additional empirical research is needed, we find evidence that “balanced” payments from competing companies may still be associated with differences in prescribing patterns.

## Supplementary Material

oyag002_Supplementary_Data

## Data Availability

The data underlying this article are available for free, public download. Open Payments: https://www.cms.gov/priorities/key-initiatives/open-payments/data/dataset-downloads. Medicare Part D Prescriber: https://data.cms.gov/provider-summary-by-type-of-service/medicare-part-d-prescribers/medicare-part-d-prescribers-by-provider-and-drug
