# Supplementary material for: How often do oncologists receive industry payments from competing companies?
Source: Oncologist. 2026 Jan 9;31(3):oyag002. doi: 10.1093/oncolo/oyag002 (PMC12875597; doi:10.1093/oncolo/oyag002)

**Table of Contents**

Supplementary Table 1…………………………………………………………………………2

Supplementary Table 2…………………………………………………………………………3

Supplementary Table 3…………………………………………………………………………4

Supplementary Table 4…………………………………………………………………………5

Supplementary Table 5…………………………………………………………………………6

Supplementary Table 6…………………………………………………………………………7

Supplementary Table 7…………………………………………………………………………8

Supplementary Table 8…………………………………………………………………………9

Supplementary Figure 1 ……………………………………………………………………...10

Supplementary Figure 2……………………………………………………………………….11

Supplementary Figure 3……………………………………………………………………….12

**Tables & Figures**

Supplementary Table 1: Mean per-physician, per-year count of 30-day prescriptions for each included drug, by physicians in the corresponding cohort. Reported both in total and separated by year. SD, standard deviation; IQR, inter quartile range. CML, chronic myeloid leukemia; EGFR, epidermal growth factor receptor inhibitors; CDK, cyclin dependent kinase 4/6 inhibitors.

|  | CML | | | EGFR | | | CDK | | |
| --- | --- | --- | --- | --- | --- | --- | --- | --- | --- |
| Drug | Dasatinib | Nilotinib | Bosutinib | Afatinib | Gefitinib | Osimertinib | Palbociclib | Ribociclib | Abemaciclib |
| Rx count, mean (SD) | 10.9 (12.3) | 10.8 (11.5) | 1.8 (6.6) | 6.9 (11.2) | 1.6 (5.4) | 23.6 (24.8) | 32.4 (21.0) | 0.7 (3.8) | 1.6 (5.8) |
| Rx count, median (IQR) | 12 (17) | 12 (17) | 0 (0) | 0 (13) | 0 (0) | 18 (20) | 27 (23.0) | 0 (0) | 0 (0) |
|  |  | | |  | | |  | | |
| Rx count, mean (SD) | Dasatinib | Nilotinib | Bosutinib | Afatinib | Gefitinib | Osimertinib | Palbociclib | Ribociclib | Abemaciclib |
| 2017 | 11.3 (12.6) | 11.5 (12.3) | 1.8 (6.0) | 8.9 (12.6) | 2.8 (7.4) | 14.4 (18.2) | 31.2 (19.0) | 0.2 (1.6) | 0 (0) |
| 2018 | 11.2 (12.4) | 11.2 (11.6) | 2.1 (7.5) | 6.8 (10.9) | 1.2 (4.3) | 24.6 (23.8) | 32.2 (19.2) | 0.7 (3.7) | 1.0 (4.2) |
| 2019 | 10.2 (11.9) | 9.8 (10.4) | 1.7 (6.1) | 5.0 (9.4) | 0.8 (3.6) | 31.9 (28.4) | 32.2 (23.9) | 1.2 (5.2) | 2.2 (6.9) |

Supplementary Table 2: Proportion of physicians who received payments for 0, 1, 2, or 3 of the competing drugs during 2017-2019. Physicians who received any general or research payment were included. GP, general payment; RP, research payment. CML, chronic myeloid leukemia; EGFR, epidermal growth factor receptor inhibitors; CDK, cyclin dependent kinase 4/6 inhibitors.

|  | CML | EGFR | CDK |
| --- | --- | --- | --- |
| N | 810 | 243 | 1756 |
| Received any GP during 2017-2019, for the indicated number of drugs, N (%) |  |  |  |
| 0 drugs | 296 (36.5) | 89 (36.6) | 526 (30.0) |
| 1 drug | 137 (16.9) | 67 (27.6) | 336 (19.1) |
| 2 drugs | 172 (21.2) | 83 (34.2) | 291 (16.6) |
| 3 drugs | 205 (25.3) | 4 (1.6) | 603 (34.3) |
|  |  |  |  |
| Received any RP during 2017-2019, for the indicated number of drugs, N (%) |  |  |  |
| 0 drugs | 763 (94.2) | 206 (84.8) | 1,538 (87.6) |
| 1 drug | 37 (4.6) | 36 (14.8) | 182 (10.4) |
| 2 drugs | 9 (1.1) | 1 (0.4) | 36 (2.1) |
| 3 drugs | 1 (0.1) | 0 (0) | 0 (0) |
|  |  |  |  |
| Received any payment (GP or RP) during 2017-2019, for the indicated number of drugs, N (%) |  |  |  |
| 0 drugs | 283 (34.9) | 79 (32.5) | 491 (28.0) |
| 1 drug | 141 (17.4) | 71 (29.2) | 336 (19.1) |
| 2 drugs | 171 (21.1) | 89 (36.6) | 313 (17.8) |
| 3 drugs | 215 (26.5) | 3 (1.6) | 616 (35.1) |

Supplementary Table 3: Proportion of physicians who received payments totaling $100 or more for 0, 1, 2, or 3 of the competing drugs during 2017-2019. GP, general payment; RP, research payment. CML, chronic myeloid leukemia; EGFR, epidermal growth factor receptor inhibitors; CDK, cyclin dependent kinase 4/6 inhibitors.

|  | CML | EGFR | CDK |
| --- | --- | --- | --- |
| N | 810 | 243 | 1,756 |
| Received any GP totaling >=$100 during 2017-2019, for the indicated number of drugs, N (%) |  |  |  |
| 0 drugs | 547 (67.5) | 133 (54.7) | 1,058 (60.3) |
| 1 drug | 179 (22.1) | 63 (25.9) | 314 (17.9) |
| 2 drugs | 76 (9.4) | 47 (19.3) | 239 (13.6) |
| 3 drugs | 8 (1.0) | 0 (0) | 145 (8.3) |
|  |  |  |  |
| Received any RP totaling >=$100 during 2017-2019, for the indicated number of drugs, N (%) |  |  |  |
| 0 drugs | 763 (94.2) | 207 (85.2) | 1,538 (87.6) |
| 1 drug | 37 (4.6) | 36 (14.8) | 182 (10.4) |
| 2 drugs | 9 (1.1) | 0 (0) | 36 (2.1) |
| 3 drugs | 1 (0.1) | 0 (0) | 0 (0) |
|  |  |  |  |
| Received payments (GP or RP) totaling >=$100 during 2017-2019, for the indicated number of drugs, N (%) |  |  |  |
| 0 drugs | 520 (64.2) | 119 (49.0) | 917 (52.2) |
| 1 drug | 196 (24.2) | 73 (30.0) | 391 (22.3) |
| 2 drugs | 80 (9.9) | 51 (21.0) | 286 (16.3) |
| 3 drugs | 14 (1.7) | 0 (0.0) | 162 (9.2) |

Supplementary Table 4: Proportion of physicians who received two or more payments for 0, 1, 2, or 3 of the competing drugs during 2017-2019. GP, general payment; RP, research payment. CML, chronic myeloid leukemia; EGFR, epidermal growth factor receptor inhibitors; CDK, cyclin dependent kinase 4/6 inhibitors.

|  | CML | EGFR | CDK |
| --- | --- | --- | --- |
| N | 810 | 243 | 1,756 |
| Received two or more GP during 2017-2019, for the indicated number of drugs, N (%) |  |  |  |
| 0 drugs | 373 (46.0) | 108 (44.4) | 755 (43.0) |
| 1 drug | 131 (16.2) | 67 (27.6) | 288 (16.4) |
| 2 drugs | 181 (22.3) | 65 (26.7) | 260 (14.8) |
| 3 drugs | 125 (15.4) | 3 (1.2) | 453 (25.8) |
|  |  |  |  |
| Received two or more RP during 2017-2019, for the indicated number of drugs, N (%) |  |  |  |
| 0 drugs | 772 (95.3) | 215 (88.5) | 1,583 (90.1) |
| 1 drug | 31 (3.8) | 28 (11.5) | 151 (8.6) |
| 2 drugs | 6 (0.7) | 0 (0) | 22 (1.3) |
| 3 drugs | 1 (0.1) | 0 (0) | 0 (0) |
|  |  |  |  |
| Received two or more payments (GP or RP) during 2017-2019, for the indicated number of drugs, N (%) |  |  |  |
| 0 drugs | 357 (44.1) | 99 (40.7) | 705 (40.1) |
| 1 drug | 139 (17.2) | 72 (29.6) | 302 (17.2) |
| 2 drugs | 182 (22.5) | 69 (28.4) | 284 (16.2) |
| 3 drugs | 132 (16.3) | 3 (1.2) | 465 (26.5) |

Supplementary Table 5: Proportion of physicians who received payments other than for Food & Beverage for 0, 1, 2, or 3 of the competing drugs during 2017-2019. GP, general payment; RP, research payment. CML, chronic myeloid leukemia; EGFR, epidermal growth factor receptor inhibitors; CDK, cyclin dependent kinase 4/6 inhibitors.

|  | CML | EGFR | CDK |
| --- | --- | --- | --- |
| N | 810 | 243 | 1,756 |
| Received any GP besides Food & Beverage during 2017-2019, for the indicated number of drugs, N (%) |  |  |  |
| 0 drugs | 668 (82.5) | 156 (64.2) | 1,353 (77.1) |
| 1 drug | 132 (16.3) | 55 (22.6) | 331 (18.8) |
| 2 drugs | 8 (1.0) | 32 (13.2) | 57 (3.2) |
| 3 drugs | 2 (0.2) | 0 (0.0) | 15 (0.9) |
|  |  |  |  |
| Received any RP during 2017-2019, for the indicated number of drugs, N (%) |  |  |  |
| 0 drugs | 763 (94.2) | 206 (84.8) | 1,538 (87.6) |
| 1 drug | 37 (4.6) | 36 (14.8) | 182 (10.4) |
| 2 drugs | 9 (1.1) | 1 (0.4) | 36 (2.1) |
| 3 drugs | 1 (0.1) | 0 (0) | 0 (0) |
|  |  |  |  |
| Received any payment (GP or RP) other than Food & Beverage during 2017-2019, for the indicated number of drugs, N (%) |  |  |  |
| 0 drugs | 641 (79.1) | 141 (58.0) | 1,201 (68.4) |
| 1 drug | 145 (17.9) | 65 (26.7) | 431 (24.5) |
| 2 drugs | 19 (2.3) | 37 (15.2) | 102 (5.8) |
| 3 drugs | 5 (0.6) | 0 (0) | 22 (1.3) |

Supplementary Table 6: Distribution of physician characteristics with respect to number of drugs for which the physician received payment, CML sub-cohort.

|  | Number of drugs for which physician received any industry payments, n (%) | | | |
| --- | --- | --- | --- | --- |
|  | 0 | 1 | 2 | 3 |
| Physician total | 283 (34.9) | 141 (17.4) | 171 (21.1) | 215 (26.5) |
| Gender |  |  |  |  |
| Woman | 69 (24.4) | 36 (25.5) | 34 (19.9) | 37 (17.2) |
| Man | 214 (75.6) | 105 (74.5) | 137 (80.1) | 178 (82.8) |
|  |  |  |  |  |
| Years in practice |  |  |  |  |
| <10 years | 1 (0.4) | 0 (0.00) | 0 (0.00) | 3 (1.4) |
| 10-20 years | 85 (30.1) | 45 (31.9) | 45 (26.5) | 58 (27.0) |
| 21-30 years | 92 (32.6) | 50 (35.5) | 63 (47.1) | 70 (32.6) |
| 30+ years | 104 (36.9) | 46 (32.6) | 62 (36.5) | 84 (39.1) |
| Unknown | 1 | 0 | 1 | 0 |
|  |  |  |  |  |
| Prescribing Volume |  |  |  |  |
| Top Quartile prescribers | 56 (19.8) | 34 (24.1) | 50 (29.2) | 66 (30.7) |
| All other prescribers | 227 (80.2) | 107 (75.9) | 121(70.8) | 149 (69.3) |

.

Supplementary Table 7: Distribution of physician characteristics with respect to number of drugs for which the physician received payment, EGFR sub-cohort.

|  | Number of drugs for which physician received any industry payments, n (%) | | | |
| --- | --- | --- | --- | --- |
|  | 0 | 1 | 2 | 3 |
| Physician total | 79 (32.5) | 71 (29.2) | 89 (36.6) | 4 (1.6) |
| Gender |  |  |  |  |
| Woman | 25 (31.6) | 18 (25.4) | 16 (18.0) | 0 |
| Man | 54 (68.4) | 53 (74.6) | 73 (82.0) | 4 (100.0) |
|  |  |  |  |  |
| Years in practice |  |  |  |  |
| <10 years | 0 | 0 | 1 | 0 |
| 10-20 years | 36 (45.6) | 28 (39.4) | 29 (32.6) | 2 (50.0) |
| 21-30 years | 20 (25.3) | 23 (32.4) | 33 (37.1) | 0 |
| 30+ years | 23 (29.1) | 20 (28.2) | 26 (29.2) | 2 (50.0) |
| Unknown | 0 | 0 | 0 | 0 |
|  |  |  |  |  |
| Prescribing Volume |  |  |  |  |
| Top Quartile prescribers | 13 (16.5) | 18 (25.4) | 30 (33.7) | 0 (0.00) |
| All other prescribers | 66 (83.5) | 53 (74.6) | 59 (66.3) | 4 (100.0) |

|  | Number of drugs for which physician received any industry payments, n (%) | | | |
| --- | --- | --- | --- | --- |
|  | 0 | 1 | 2 | 3 |
| Physician total | 491 (28.0) | 336 (19.1) | 313 (17.8) | 616 (35.1) |
| Gender |  |  |  |  |
| Woman | 193 (39.3) | 158 (47.0) | 122 (39.0) | 173 (28.1) |
| Man | 298 (60.7) | 178 (53.0) | 191 (61.0) | 443 (71.9) |
|  |  |  |  |  |
| Years in practice |  |  |  |  |
| <10 years | 6 (1.2) | 5 (1.5) | 2 (0.6) | 6 (1.0) |
| 10-20 years | 178 (36.3) | 104 (31.0) | 95 (30.6) | 184 (29.9) |
| 21-30 years | 155 (31.6) | 110 (32.7) | 101 (32.5) | 193 (31.3) |
| 30+ years | 151 (30.8) | 117 (34.8) | 113 (36.3) | 233 (37.8) |
| Unknown | 1 | 0 | 2 | 0 |
|  |  |  |  |  |
| Prescribing Volume |  |  |  |  |
| Top Quartile prescribers | 79 (16.1) | 103 (30.7) | 97 (31.0) | 161 (26.1) |
| All other prescribers | 412 (83.9) | 233 (69.3) | 216 (69.0) | 455 (73.9) |

Supplementary Table 8: Distribution of physician characteristics with respect to number of drugs for which the physician received payment, CDK sub-cohort.

Supplementary Figure 1: CONSORT diagram detailing cohort derivation. RCC, renal cell carcinoma; CML, chronic myeloid leukemia; EGFR, epidermal growth factor receptor inhibitors; ALK, anaplastic lymphoma kinase inhibitors; PARP, poly ADP ribose polymerase inhibitors; CDK, cyclin dependent kinase 4/6 inhibitors.


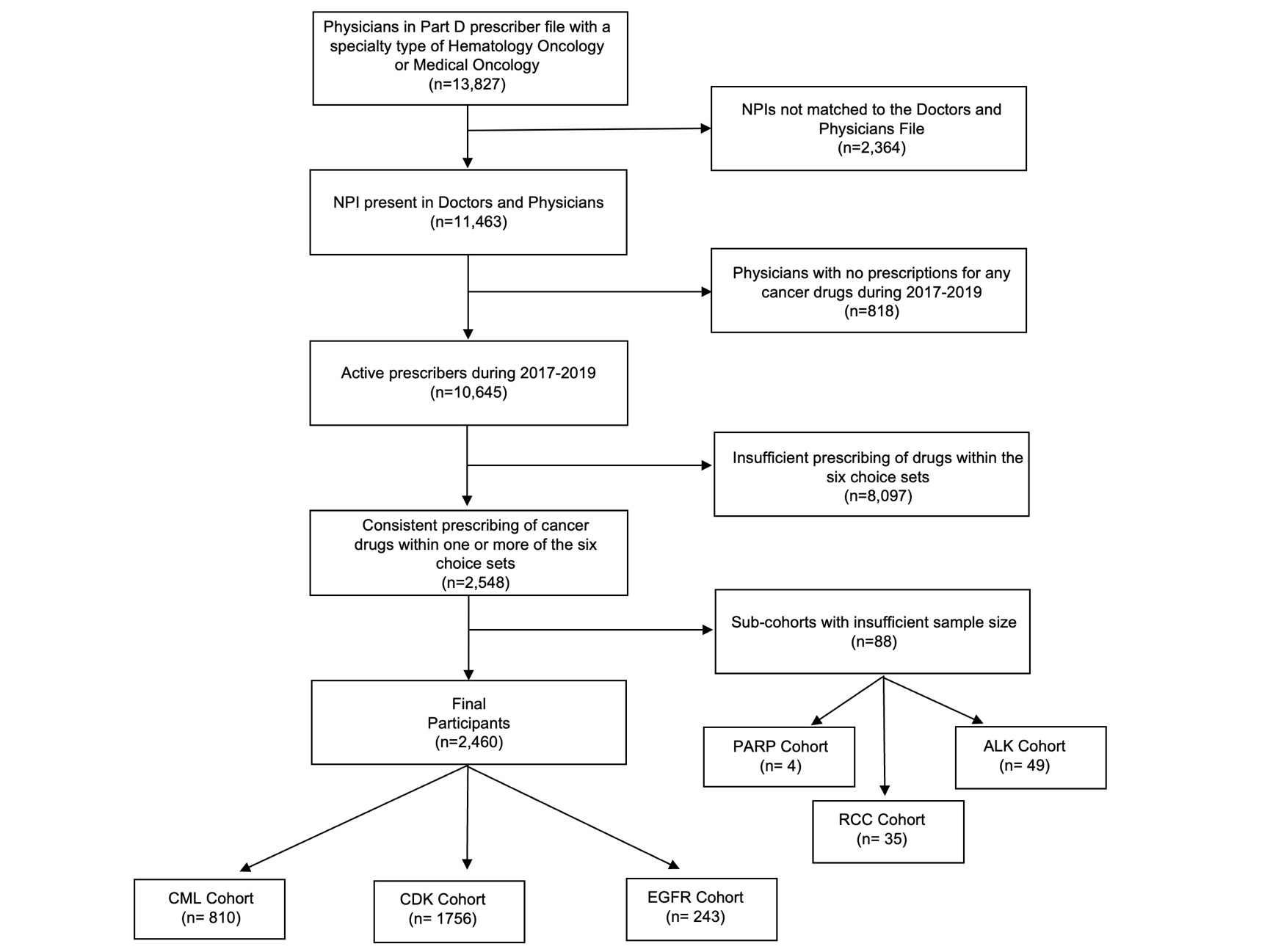


Supplementary Figure 2: Distribution of industry payments received by physicians within each sub-cohort related to the 3 drugs of interest across 2017-2019. General and research payments are included in payment totals. CML, chronic myeloid leukemia; EGFR, epidermal growth factor receptor inhibitors; CDK, cyclin dependent kinase 4/6 inhibitors.


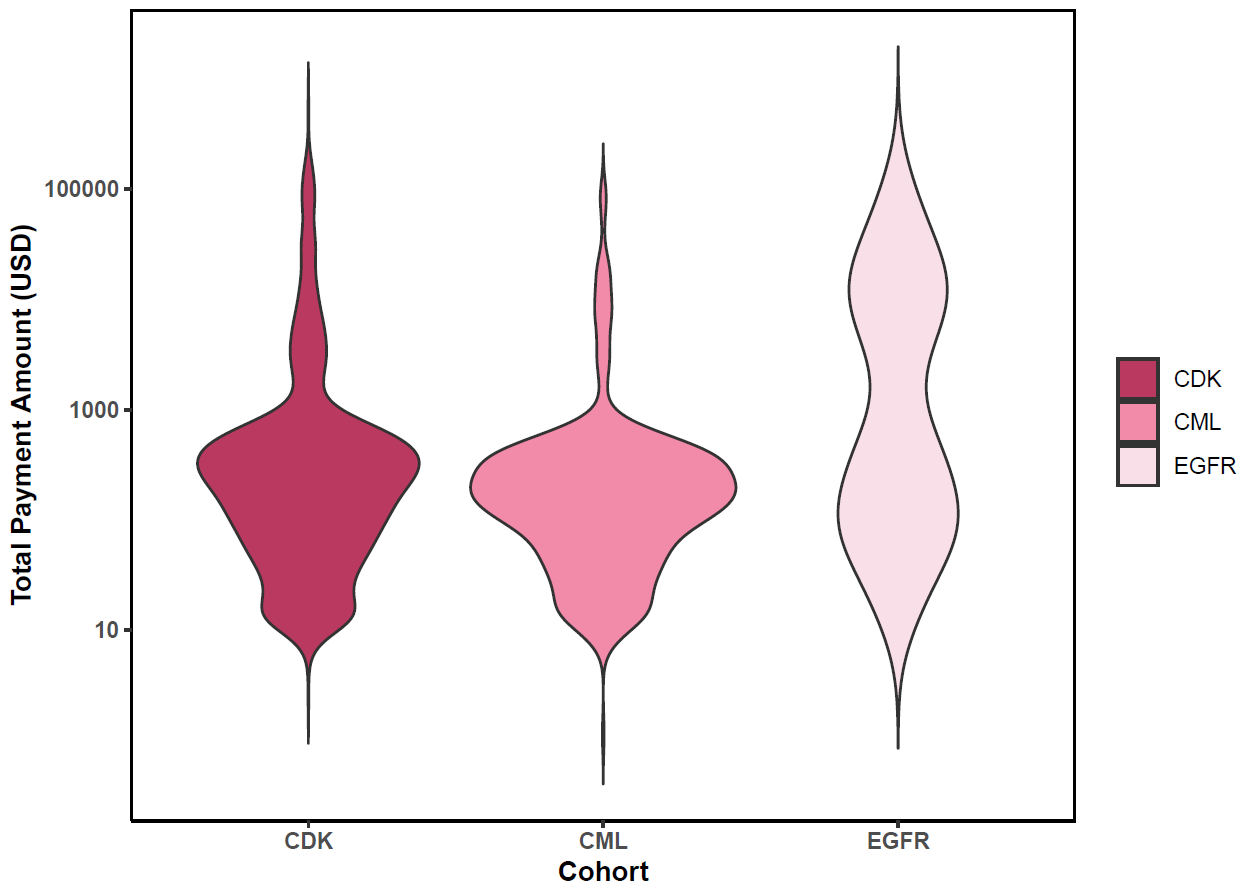


Supplementary Figure 3: Histogram showing the number of physician-cohort pairs that received total payment amounts within the indicated ranges. Payment total is inclusive of both general and research payments from 2017-2019 for drugs in the corresponding choice set for each sub-cohort.


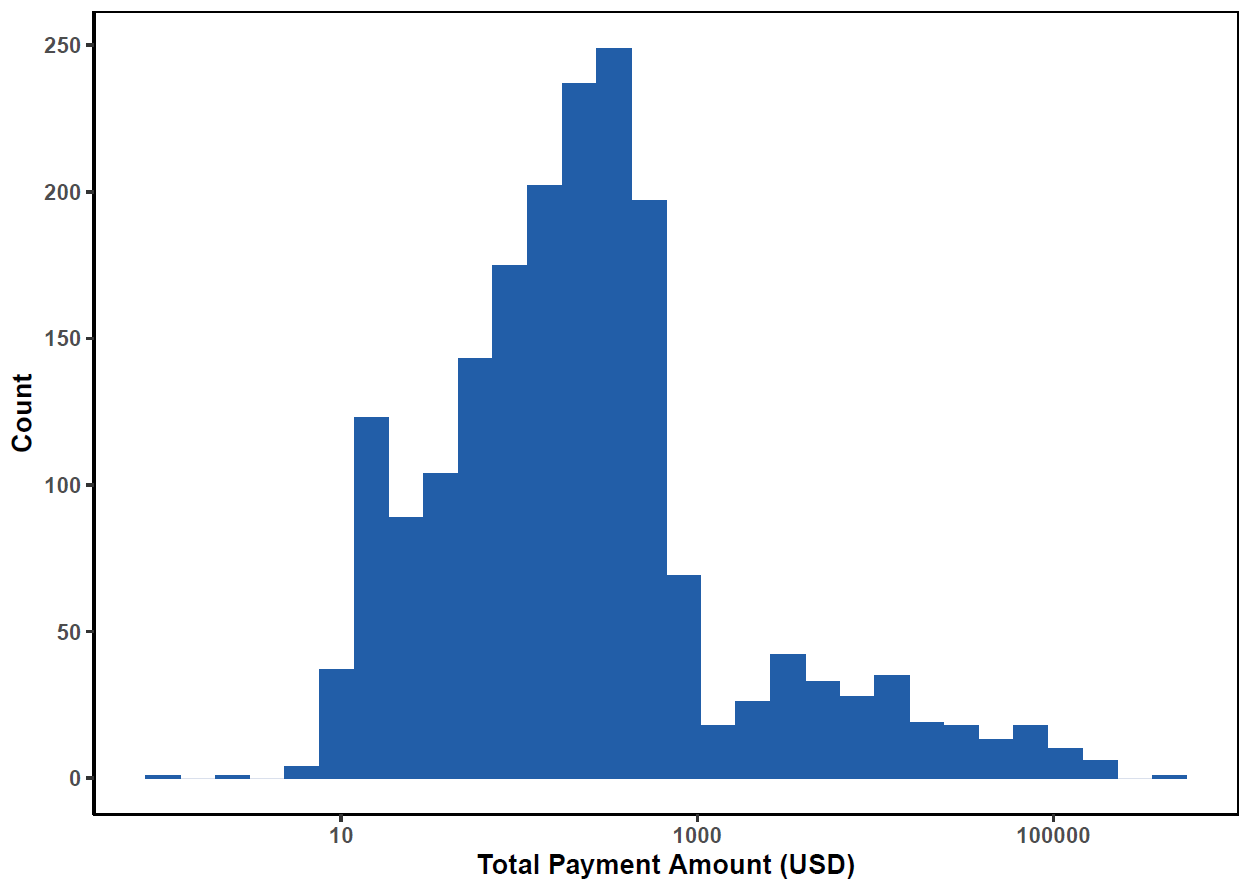

Supplement: oyag002_Supplementary_Data [file oyag002_supplementary_data.docx]
